# Development of Hybrid DSPC:DOPC:P(OEGMA_950_-DIPAEMA) Nanostructures: The Random Architecture of Polymeric Guest as a Key Design Parameter

**DOI:** 10.3390/polym15091989

**Published:** 2023-04-22

**Authors:** Efstathia Triantafyllopoulou, Dimitriοs Selianitis, Natassa Pippa, Maria Gazouli, Georgia Valsami, Stergios Pispas

**Affiliations:** 1 Section of Pharmaceutical Technology, Department of Pharmacy, School of Health Sciences, National and Kapodistrian University of Athens, Panepistimioupolis Zografou, 15771 Athens, Greece; efstrian@pharm.uoa.gr (E.T.); natpippa@pharm.uoa.gr (N.P.); valsami@pharm.uoa.gr (G.V.); 2 Theoretical and Physical Chemistry Institute, National Hellenic Research Foundation, 48 Vassileos Constantinou Avenue, 11635 Athens, Greece; dimitrissel404@gmail.com; 3 Laboratory of Biology, Department of Basic Medical Science, School of Medicine National and Kapodistrian, University of Athens, 11527 Athens, Greece; mgazouli@med.uoa.gr

**Keywords:** hybrid nanoparticles, lipid, polymer, pH-responsive nanocarriers, thermoresponsive nanocarriers, P(OEGMA-co-DIPAEMA), random architecture, lipid to polymer ratio, DSC

## Abstract

Hybrid nanoparticles have gained a lot of attention due to their advantageous properties and versatility in pharmaceutical applications. In this perspective, the formation of novel systems and the exploration of their characteristics not only from a physicochemical but also from a biophysical perspective could promote the development of new nanoplatforms with well-defined features. In the current work, lipid/copolymer bilayers were formed in different lipid to copolymer ratios and examined via differential scanning calorimetry as a preformulation study to decipher the interactions between the biomaterials, followed by nanostructure preparation by the thin-film hydration method. Physicochemical and toxicological evaluations were conducted utilizing light scattering techniques, fluorescence spectroscopy, and MTS assay. 1,2-dioctadecanoyl-sn-glycero-3-phosphocholine (DSPC) and 1,2-dioleoyl-sn-glycero-3-phosphocholine (DOPC) in different weight ratios were the chosen lipids, while a linear random copolymer with pH- and thermoresponsive properties comprised of oligo (ethylene glycol) methyl ether methacrylate (OEGMA) and 2-(diisopropylamino) ethyl methacrylate (DIPAEMA) in different ratios was used. According to our results, non-toxic hybrid nanosystems with stimuli-responsive properties were successfully formulated, and the main parameters influencing their overall performance were the hydrophilic/hydrophobic balance, lipid to polymer ratio, and more importantly the random copolymer topology. Hopefully, this investigation can promote a better understanding of the factors affecting the behavior of hybrid systems.

## 1. Introduction

Hybrid nanoparticles have attracted the interest of the scientific community in recent years, especially as drug delivery systems [1,2,3,4,5,6]. This is attributed especially to their ability to maintain the biophysical properties of all the components. In this manner, hybrid nanoplatforms reveal unique properties, simultaneously limiting the disadvantages of the individual biomaterials [7]. Among other hybrid nanocarriers, lipid/polymer nanostructures can be distinguished. The biocompatibility of liposomes and the physical stability of polymeric nanoparticles are well established [8,9,10,11]. By exploiting their advantages and modifying their characteristics, they can be utilized as ideal nanocarriers for modified release and the delivery of challenging molecules, such as active pharmaceutical ingredients (APIs) of low solubility or fragile macromolecules (e.g., nucleic acids, proteins, and peptides) [10,11]. For example, chitosan/lipid shell–core structured hybrid systems were developed for the oral administration of poorly soluble indomethacin with controlled release and mucoadhesive properties, while an innovative inhalable formulation was developed via the Quality by Design approach comprised of a lipid/polymer nanocarrier simultaneously incorporating an anticancer API and a nucleic acid for the synergistic treatment of lung cancer [12,13]. In comparison to pure nanoparticles, the hybrid systems offer the asset of well-defined properties and robust structures, being promising in novel treatment [8,11,12,13].

Additionally, the smart nanoplatforms that perceive and respond to an internal or external stimulus have created new pathways in therapy for spatiotemporal release. There is extensive literature for advanced drug delivery systems which exhibit stimuli-responsive characteristics, especially for oncotherapy, whereas several release triggers exist, including light, pH, redox, and heat [8,14,15,16,17]. Focusing on pH-sensitive nanocarriers, their applications involve the site-specific delivery of molecules due to differentiation in environmental pH by preferring to release their cargo in specific tissues (e.g., vagina) or escaping organelles (e.g., endosomes) or preventing degradation of the incorporated molecules in the gastric environment. pH responsiveness is valuable and extensively used in targeting pathological sites as well, especially tumors [14,18]. pH-responsive polymers are ideal excipients for the development of such delivery systems. Apart from cross-linking and cleavable groups, another strategy is alteration in hydrophilicity. Namely, pH-responsive polymers could have ionizable groups, which at different pH values are protonated or deprotonated. Hence, the basic principle is the re-assembly of the polymeric chains in an aqueous medium due to pH changes depending on the polymer pKa [18,19]. Some polymers that are widely used for this purpose in drug delivery design are chitosan, poly(N-isopropylacrylamide) (PNIPAM), poly(caprolactone) (PCL), poly(2-(dimethylamino)ethyl methacrylate) (PDMAEMA), poly(propyl acrylic acid) (PPAA), and poly(methacrylic acid) (PMAA) [19]. On the other hand, thermoresponsive nanostructures are also promising for targeted release, especially combined with hyperthermia in cancer treatment [14,15,16,17,20]. Temperature-responsive polymers could differentiate their morphology according to heat variations in an aqueous solution. Specifically, in response to temperature changes different interactions of the polymeric chains with water molecules take place, leading to a nanoparticulate morphology, a different conformation, or dis-assembly depending on intrinsic properties of the polymer, particularly its critical solution temperature (CST, lower or upper) [17,20]. PNIPAAm copolymers and marketed available Pluronic F-127 are among the most popular thermoresponsive excipients [14,15,16,17]. In general, synthetic polymers are advantageous due to their tailor-made nature, fine-tuning their properties and stimuli responsiveness according to intended needs.

The aim of this study is to design and develop novel hybrid nanostructures with stimuli-responsive properties as potential nanocarriers for modified drug release and examine them from the scope of the parameters affecting their thermodynamic and physicochemical behavior. For this purpose, lipid/copolymer systems were formed utilizing two commonly used phospholipids, DSPC and DOPC, a solid and a liquid at ambient temperature, respectively, and a pH- and thermo- responsive copolymer, particularly the P(OEGMA-co-DIPAEMA) random copolymer in two different % ratios of comonomers. POEGMA is a thermoresponsive hydrophilic homopolymer with “non-fouling” properties. Its cloud point (T_cp_) is proportional to the side-chain length, tunable for copolymers and for POEGMA_500_ at around 90 °C [21,22,23,24,25]. The homopolymer of PDIPAEMA is a thermo- and pH-responsive polymer [21,26,27]. According to the literature, the POEGMA homopolymer as well as P(OEGMA-co-DIPAEMA) random copolymers are well tolerated and mainly non-toxic [21,22]. The formulation protocol that was chosen was the thin-film hydration one, as it is a well-established method to prepare colloidal dispersions [28]. The protocol was the same for all the examined systems, and no process parameter was changed to investigate which belongs to the critical ones. Preparing the hybrid systems in the exact same manner is a way to ensure that the different characteristics of the systems are owed exclusively to the alteration of designing factors (e.g., lipid to polymer ratio, lipid composition, etc.) and not to modifications of the procedure. The rational design of drug delivery systems includes the investigation of their physical properties as well as their optimization to form nanocarriers with desired characteristics and pharmacokinetic profiles [29]. In this regard, a gamut of techniques was used to characterize the hybrid systems from the standpoint of the thermotropic behavior of the biomaterials and the physicochemical and toxicological properties of the nanostructures. To the best of our knowledge, this is the first report elucidating the interactions between the P(OEGMA-co-DIPAEMA) random copolymer, DSPC, and DOPC lipids, as well as highlighting the contribution of random architecture to unique properties of lipid/polymer hybrid systems.

## 2. Materials and Methods

### 2.1. Materials

The lipids used for the preparation of hybrid systems were 1,2-dioctadecanoyl-sn-glycero-3-phosphocholine (DSPC) and 1,2-dioleoyl-sn-glycero-3-phosphocholine (DOPC) (Table S1), which were purchased from Lipoid GmbH. Chloroform and all other reagents used were of analytical grade and purchased from Sigma–Aldrich Chemical Co. (St. Louis, MO, USA). P(OEGMA-co-DIPAEMA) statistical (random) copolymers were synthesized by RAFT polymerization methodology and their characteristics are summarized in Table 1. There is additional information about their synthetic route and characterization in the Supplementary Material (Scheme S1, Figures S1 and S2).

### 2.2. Methods

#### 2.2.1. Differential Scanning Calorimetry

DSC experiments were conducted on an 822e Mettler-Toledo (Schwerzenbach, Switzerland) calorimeter. The calibration was performed with pure indium (T_m_ = 156.6 °C) and water. The hybrid bilayers were composed of either DSPC:P(OEGMA_950_-co-DIPAEMA) (37 or 70% PDIPAEMA) or DSPC:DOPC:P(OEGMA_950_-co-DIPAEMA) (37 or 70% PDIPAEMA) at different lipid to polymer weight ratios, while the chosen DSPC:DOPC weight ratio was 9:1 or 4:6. The systems were dissolved in chloroform and the solvent was removed first by slow evaporation and then under vacuum overnight. The dried material was weighed into sealed aluminum crucibles of 40 μL capacity and hydrated using HPLC-grade water or in some experiments HCl 0.1 N. An empty aluminum crucible was used as reference. Prior to measurements the crucibles were heated at a temperature that exceeds the transition of DSPC (55 °C) to ensure equilibration. Two heating–cooling cycles were performed: 20 to 60 °C at 5 °C/min scanning rate. The results were evaluated during the second cooling and heating cycle and the errors were based on at least three replicates. All samples were scanned until identical curves were obtained. The calorimetric parameters were calculated with Mettler-Toledo STARe software.

#### 2.2.2. Preparation of Lipid/Copolymer Hybrid Systems

Different hybrid colloidal systems were prepared by the thin-film hydration method at a concentration equal to C = 5 mg/mL in different lipid to polymer weight ratios, namely 9:1, 7:3, and 5:5. They consisted of DSPC or DSPC:DOPC 9:1 (weight ratio) as the lipidic part and the linear random copolymer P(OEGMA_950_-DIPAEMA) in different proportions of PDIPAEMA (37% PDIPAEMA or copolymer P(OEGMA_950_-DIPAEMA)-1 and 70% PDIPAEMA or copolymer P(OEGMA_950_-DIPAEMA)-2), namely DSPC:P(OEGMA_950_-DIPAEMA)-1 (9:1 weight ratio), DSPC:P(OEGMA_950_-DIPAEMA)-1 (7:3 weight ratio), DSPC:P(OEGMA_950_-DIPAEMA)-1 (5:5 weight ratio), DSPC:P(OEGMA_950_-DIPAEMA)-2 (9:1 weight ratio), DSPC:P(OEGMA_950_-DIPAEMA)-2 (7:3 weight ratio), DSPC:P(OEGMA_950_-DIPAEMA)-2 (5:5 weight ratio), DSPC:DOPC:P(OEGMA_950_-DIPAEMA)-1 (9:1 weight ratio), DSPC:DOPC:P(OEGMA_950_-DIPAEMA)-1 (7:3 weight ratio), DSPC:DOPC:P(OEGMA_950_-DIPAEMA)-1 (5:5 weight ratio), DSPC:DOPC:P(OEGMA_950_-DIPAEMA)-2 (9:1 weight ratio), DSPC:DOPC:P(OEGMA_950_-DIPAEMA)-2 (7:3 weight ratio), and DSPC:DOPC:P(OEGMA_950_-DIPAEMA)-2 (5:5 weight ratio). Briefly, suitable amounts of each component were dissolved in chloroform and then transferred into a round-bottom flask. The flask was inserted into a rotary evaporator (Rotavapor) and vacuum was applied at 45 °C for 30 min in order for the organic solvent to be evaporated and a thin film to be formed on the inner walls of the flask. The film was hydrated with HPLC-grade water by slowly spinning the flask in a water bath for 1 h at a temperature above the phase transition temperature of the systems according to DSC results (see Section 3.1). Afterwards, the colloidal suspensions were subjected to probe sonication of two 5 min sonication cycles interrupted by a resting period of 5 min.

#### 2.2.3. Light Scattering Methods

The hydrodynamic radius (R_h_) of the prepared hybrid systems, the polydispersity index (PDI), and the scattering light intensity (I) along with the radius of gyration (R_g_), when necessary, were measured by dynamic and static light scattering (DLS and SLS, respectively) techniques. For these studies, 50 μL of dispersion was diluted with 2 mL of HPLC-grade water. Dynamic light scattering measurements were carried out at a fixed scattering angle of 90° and at different temperatures (25, 37, and 60 °C), allowing for 5 min equilibration, whereas HCl 0.1 N and phosphate buffer saline (PBS) were also used as dilution media, and these samples were measured at ambient temperature. Apart from aqueous medium the other two different pH media were chosen to stimulate physiological conditions, where 37 °C corresponds to body temperature and 60 °C is a temperature above the main phase transition of the systems, where the membrane should be in the liquid crystalline state according to DSC results (see Section 3.1). Referring to SLS experimental conditions the angular range was 30°–150°, with temperature fixed at 25 °C. Toluene was used as the calibration standard, while profiles were analyzed by Zimm and Guinier models using the software available by the manufacturer. Measurements were performed on an ALV/CGS-3 Compact Goniometer System (ALV GmbH, Germany) and analyzed by the CONTIN algorithm. This set up includes a He-Ne 22 mW laser source, a compact goniometer system with an avalanche photodiode detector interfaced with an ALV/LSE-5003 module, an ALV-5000/EPP multi-tau digital photon correlator, and a Polyscience model 9102 bath circulator for temperature control.

#### 2.2.4. Fluorescence Spectroscopy

The fluorescence spectra were collected in order to gather information on the internal environment of the hybrid nanosystems and specifically on their micropolarity characteristics (NanoLog Fluorometer spectrometer by Horiba Jobin Yvon). All experiments were conducted at ambient temperature. The excitation wavelength was λ = 335 nm for pyrene and emission spectra were recorded in the region of 355–630 nm, with an increment of 1 nm, using an integration time of 0.5 s. Slit openings of 2 nm were used for both the excitation and the emitted beams. The insertion protocol in the sample cell followed was 1 mL of sample amalgamated with 1 µL of probe. Pyrene was utilized as the hydrophobic probe, while the dispersions containing the probe were left for a 24 h rest period in the dark at room temperature. The pyrene monomer fluorescence has five predominant peaks. The intensity ratio of peak 1 to peak 3, I_1_/I_3_, was used as a measure of the micropolarity of the medium surrounding the probe.

#### 2.2.5. In Vitro Toxicity

HEK293 cells were cultivated using a DMEM high-glucose culture medium (provided by BioSera) containing 10% FBS, 2 mmol/L glutamine, 100 IU/mL penicillin, and 100 µg/mL streptomycin. The cultivation took place at a fixed temperature of 37 °C. The medium was replaced every 48 h and the cells were passaged on a weekly basis using the trypsin/EDTA method. After reaching a sufficient confluency, cells were transferred to a 96-well plate and 5000 cells/well were seeded. Incubation took place using a steri-cycle CO_2_ incubator (HEPA Class 100, Thermo Electron Corporation^®^, Franklin, MA, USA) and cell viability was evaluated utilizing a microscope. The MTS assay was performed (CellTitre 96 R Aqueous MTS, Promega) to quantify the reduction in viability due to exposure to hybrid nanostructures. The main principle of this protocol is based on the action of mitochondrial dehydrogenase enzymes that consume NAD(P)H to reduce a tetrazolium compound (MTS) to formazan. The concentration of the latter is proportional to the number of living cells and is calculated via an absorbance reading at 490 nm using a microplate spectrophotometer (SPECTROstarNano, BMG LABTECH, Ortenberg, Germany). The different concentrations of each hybrid system to which cells were exposed ranged between 25–500 µg/mL. Incubation time was set at 24 h. Furthermore, three types of controls were used: a positive (cells with culture medium were not exposed to copolymer aggregates), a negative (copolymer aggregates without cells), and a background control (culture medium alone). Absorbances obtained were normalized with respect to the untreated control cultures to calculate changes in cell viability. All procedures were conducted in a sterile environment and all experiments were replicated twice to ensure reproducibility.

## 3. Results and Discussion

### 3.1. Preformulation Studies of Lipid/Polymer Hybrid Bilayers

#### 3.1.1. The Random Copolymer/Phospholipid Interactions in DSPC:P(OEGMA_950_-co-DIPAEMA) Hybrid Bilayers

**Figure 1:**
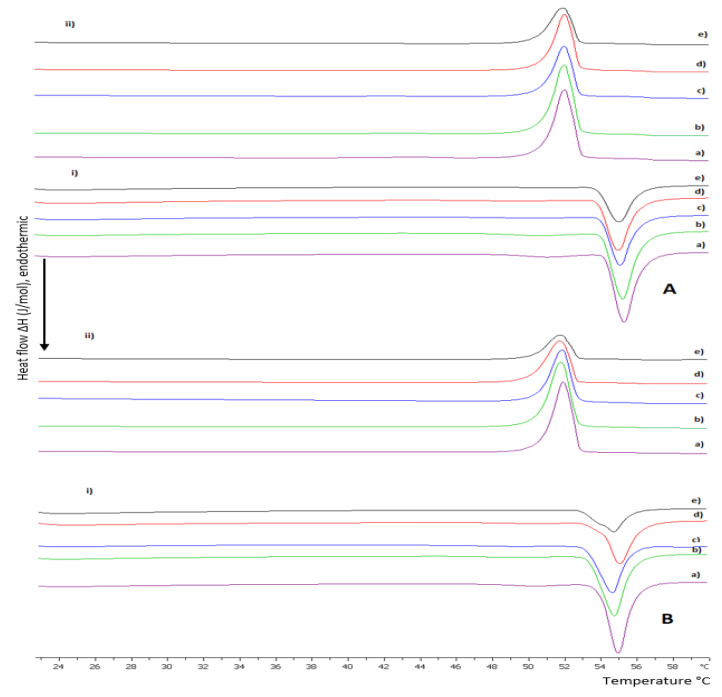
DSC thermograms during (**i**) heating and (**ii**) cooling of (**A**) DSPC:P(OEGMA_950_-DIPAEMA)-1 and (**B**) DSPC:P(OEGMA_950_-DIPAEMA)-2 hybrid bilayers in different weight ratios: (a) 9:1, (b) 8:2, (c) 7:3, (d) 6:4, and (e) 5:5 hydrated in HPLC-grade water (pH 7.0).

Τhe thermotropic behavior of DSPC lipid bilayers with the incorporation of amphiphilic random copolymers in different weight ratios is presented in Figure 1 and in Tables S2 and S3. The DSPC lipid is a zwitterionic phosphatidylcholine with long and linear acyl chains, which are saturated and even-chained as well (Table S1). During heating, lipid bilayers comprising pure DSPC show anticipated thermotropic performance despite thorough polymorphism, which involves three thermal events, sub-transition, pretransition, and main transition, corresponding to the conversion from an ordered state of the phospholipids to a disordered one, where the sub-transition is not usually visible [30,31,32]. Contrarily, amphiphilic copolymers self-assemble in aqueous media above a concentration threshold or due to a stimulus into various intra- or inter-molecular conformations depending on their composition, hydrophobic to hydrophilic ratio, architecture, and degree of polymerization [33,34]. The linear random copolymer P(OEGMA_950_-co-DIPAEMA that we used in different hydrophobic to hydrophilic ratios is illustrated in Table 1 and has stimuli-responsive properties, as mentioned before, due to the DIPAEMA segment. POEGMA is comprised of a hydrophobic main chain and PEG side chains. Its structure is comb-shaped, while its side chains dehydrate by increasing the temperature in aqueous media, leading to self-assembly and eventually aggregation [22,23,24,25]. However, in this temperature range the OEGMA segment is highly hydrophilic and cannot display its thermosensitivity due to its LCST, which is above 90 °C according to bibliographic data [22,23,24,25]. Thermo- and pH- responsive PDIPAEMA is hydrophobic above pH 6.2 with a T_cp_ around 28 °C [21,27].

According to the literature, the pure DSPC bilayer exhibits a main sharp endothermic peak at approximately 55 °C accompanied by a low enthalpic pretransition peak [30,31,35,36]. The addition of P(OEGMA_950_-DIPAEMA)-1 in increasing amounts into the DSPC bilayer (DSPC:copolymer 9:1, 8:2, 7:3, 6:4, and 5:5 weight ratios) did not drastically change the thermodynamic characteristics of the system, as observed in Figure 1 and Table S2. Specifically for the system 9:1, the main transition shows similar results to pure DSPC (T_onset_ = 54.7 °C, T_m_ = 55.2 °C, and ΔT_1/2_ = 1.16 °C). The pretransition event is also present at 50.8 °C. In general, the increase in the copolymer amount leads to a decrease in T_onset_ (the lowest value is 53.7 °C for the 5:5 system) and T_m_ (the lowest value is 54.8 °C for the 6:4 system) and a rise in peak width (the highest value is 1.36 °C for the 5:5 system). However, these differences are negligible, indicating that the copolymer does not much affect the arrangement of hydrophobic tails and that there is good cooperativity between the components. The moderate influence on the lipid bilayer by highly water-soluble polymers has been mentioned before [37]. Even though the small proportion of the polymer and the random architecture of the monomers favored the maintenance of the pretransition until 20% weight P(OEGMA_950_-DIPAEMA)-1, above 30% weight of the copolymer this thermal event is absent. The impact of the copolymer is more obvious considering the enthalpy decreasing trend (from 366 J/mol for 9:1 system to 223 J/mol for 5:5 system) and the loss of the ripple phase. It is interesting that the enthalpy for the system 8:2 is slightly higher than 9:1 and afterwards is dropped 100 J/mol lower for the 7:3 system accompanied by the absence of pretransition. These observations could be attributed to the increase in the distance of the lipids due to either repulsive forces between the hydrophilic chains of the copolymer or steric hindrance by the bulky tertiary amine group modulating bilayer mechanical properties and hence decreasing van der Waals interactions between hydrocarbon chains and reorienting the polar head groups [37,38,39]. Taking into consideration the chemical structure and the size of the copolymer, it could be assumed that the OEGMA long hydrophilic side chains would be in the outer aqueous region interacting with the solvent and stabilizing the bilayer due to hydrogen bonding and steric effects, whereas the hydrophobic main chain of POEGMA and the hydrophobic DIPAEMA comonomers—with the partially deprotonated nitrogen group—could be anchored into the bilayer through hydrophobic interactions and located probably at the interface of the hydrophobic core layer and the polar head groups, altering the polar region of the bilayer [37]. This should be further investigated in order to be confirmed. During the cooling process, the main transition peak seems to be reversible for all the systems, with a reasonable hysteresis of 3 °C (Table S3) [40]. The main event is starting at an average value of 52.8 °C and is centered at 51.8 °C. The peak width and the enthalpy values are a little higher for all the systems, implying the necessity for more energy to achieve the crystallization event [39].

Referring to the incorporation of P(OEGMA_950_-DIPAEMA)-2 in the DSPC bilayer, its thermotropic behavior is similar to P(OEGMA_950_-DIPAEMA)-1, a reasonable fact due to the copolymer architecture. The expression of strong hydrophobic or hydrophilic characteristics is not necessary for amphiphilic statistical copolymers compared to block copolymers because of the random pattern of monomers [33]. Despite similar trends in the calorimetric parameters, the addition of P(OEGMA_950_-DIPAEMA)-2 led to the absence of the pretransition peak earlier and to a relevant less cooperative hybrid system in the weight ratio of 5:5 (ΔT_1/2_ = 1.62 °C). In fact, this peak shows a barely distinguishable shoulder, which is non-reversible according to the cooling scan (Figure 1). The entirely different hydrophobic to hydrophilic ratio of the copolymer may be the reason for these differences, as it is a quite important parameter for random copolymers [33]. Due to the chemical structure of DIPAEMA, which influences both polar and apolar groups, it is extremely likely that it will be found at the bilayer’s interface. This might lead to a breakdown of the phospholipids, phase separation, and co-existence of polymer-rich and polymer-poor domains [41,42].

Overall, our calorimetric results indicate that there is not an obvious influence of T melting of the copolymers into the bilayer. However, the presence of copolymers leads to changes in the thermotropic characteristics of the lipid bilayer not as much in the main transition temperature, but especially in the pretransition and enthalpy, reflecting a different conformation of the phospholipids due to the incorporation of the copolymer into the bilayer.

#### 3.1.2. The Influence of pH on DSPC:P(OEGMA_950_-co-DIPAEMA) Hybrid Bilayers

**Figure 2:**
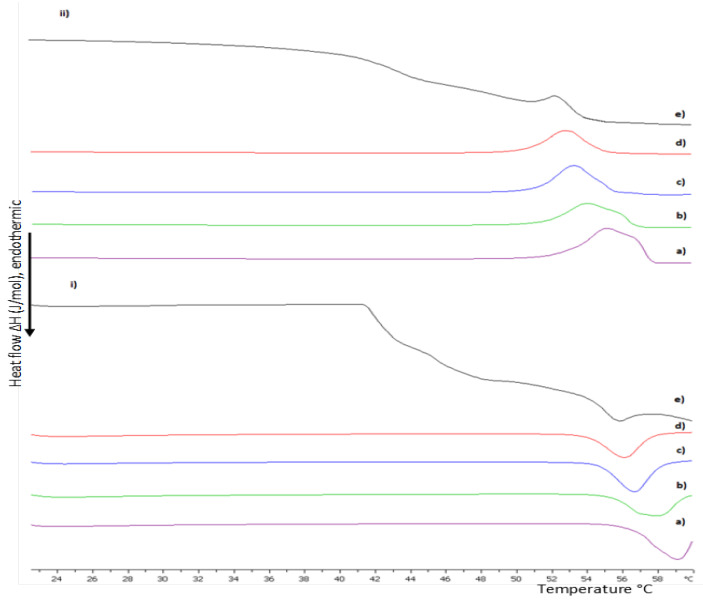
DSC thermograms during (**i**) heating and (**ii**) cooling of DSPC:P(OEGMA_950_-DIPAEMA)-1 hybrid bilayers in different weight ratios: (a) 9:1, (b) 8:2, (c) 7:3, (d) 6:4, and (e) 5:5 dispersed in HCl 0.1 N (pH 1.2).

**Figure 3:**
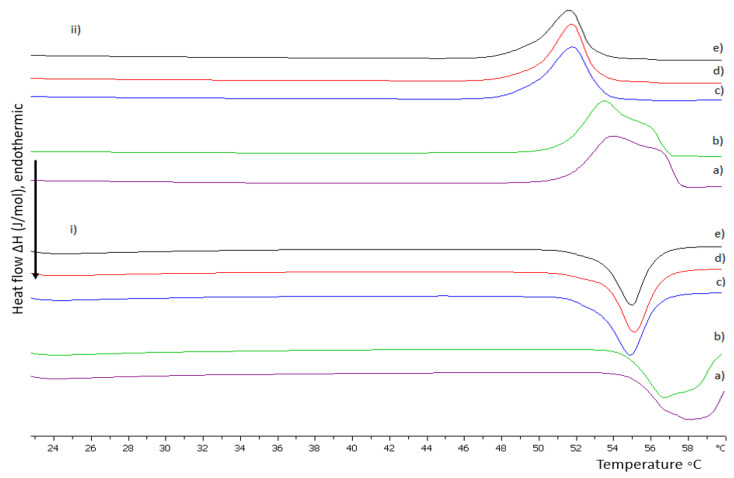
DSC thermograms during (**i**) heating and (**ii**) cooling of DSPC:P(OEGMA_950_-DIPAEMA)-2 hybrid bilayers in different weight ratios: (a) 9:1, (b) 8:2, (c) 7:3, (d) 6:4, and (e) 5:5 dispersed in HCl 0.1 N (pH 1.2).

Considering the pH-responsive nature of PDIPAEMA, DSC experiments were conducted in an acidic environment as well. The calorimetric results are summarized in Tables S4 and S5 and depicted in Figure 2 and Figure 3. Generally, DIPAEMA at pH values lower than its pKa (6.2) is protonated, leading to electrostatic interactions with polar head groups and rearrangement which is reflected by the absence of pretransition for both copolymers even at a 9:1 weight ratio [37,39]. The different orientation of the P(OEGMA_950_-DIPAEMA)-1 in the lipid bilayer results in unique thermotropic characteristics compared to the aqueous medium, as can be seen in Table S4. An increase in the temperature at which the thermotropic event starts (T_on_) and T_m_ occurs, as well as in the ΔΤ_1/2_ value, is accompanied by a decrease in enthalpy. These findings indicate that copolymer entry and exit points limit lipid mobility and lead to a more compact and rigid bilayer, which does not favor conformational freedom [43]. This thermodynamic pattern is obvious at all weight ratios. A possible explanation for the increased T_m_ and thus membrane rigidity includes more intra- and inter-molecular hydrogen bonding due to more hydrophilic segments [44]. In general, by increasing the polymer content in the bilayer there are no calorimetric trends like those we observed in aqueous medium. In fact, ΔH is increased until a threshold of 30% weight of the copolymer and afterwards is dropped, while the ΔΤ_1/2_ values exhibit no pattern, as can be observed in Table S4. Keeping in mind that phosphatidylcholines are affected by such a low pH value with DSPC showing a quite high melting temperature (T_m_ = 64 °C) [31], the T_m_ value for the hybrid system at a 9:1 weight ratio is lower and observed at 59 °C, whereas the increase in the copolymer content is accompanied by a decreasing trend for the T_m_ parameter. Hybrid bilayers of 9:1 and 8:2 correspond to asymmetric and wide peaks, implying heterogeneity as mentioned before. This behavior is absent at 7:3 and 6:4 weight ratios. However, at an equal weight of lipid to polymer even new metastable phases are created. The polar and highly hydrophilic copolymer might lead to disconformation and a rearrangement of the lipids. The low enthalpic value may be an indication of two or more different microstructures or a macroscopic phase separation [39,41,42].

Even though in an acidic pH the incorporation of P(OEGMA_950_-DIPAEMA)-2 shows similar behavior to P(OEGMA_950_-DIPAEMA)-1 as far as calorimetric trends are concerned, the data are pointing out an interesting outcome. The DIPAEMA-rich P(OEGMA_950_-DIPAEMA)-2 reflects a more obvious lipid to polymer ratio dependent behavior. In particular, a copolymer proportion of 10 or 20% weight causes immiscibility in the bilayer via wide curves and shoulder formation, as is clearly illustrated in Figure 2. In fact, disturbance of the membrane is further supported by low cooperativity (ΔΤ_1/2_ = 3.38 and 3.29 °C, respectively) [45]. On the other hand, bilayers with 30% weight of the copolymer or more correspond to similar calorimetric characteristics in the aqueous medium, apart from enthalpy which is higher. This enthalpic stabilization, highlighting more interactions, is due to favorable hydrogen bonds overcoming ionic repulsive forces between protonated chemical groups [31]. It is worth mentioning that the calorimetric characteristics are improved for the 5:5 system over its heating profile in HPLC-grade water, where a shoulder was observed (Figure 1). In our opinion, the dominance of polar DIPAEMA in P(OEGMA_950_-DIPAEMA)-2 and the random architecture may be responsible for the lipid to polymer ratio dependent behavior. In any case, this dual performance should be further examined and correlated to the release mechanism of these systems, as a different stability in the stomach environment is anticipated.

#### 3.1.3. The Influence of Unsaturated DOPC in Hybrid Bilayers

The addition of the DOPC lipid in different amounts intended to decipher how the fluidization effect influences the hybrid system performance. The DOPC phospholipid is linear and even-chained, with the same length as DSPC (18 carbon atoms )). However, DOPC has cis-type unsaturated aliphatic chains (9Z) in comparison to DSPC (Table S1) [31,36]. The existence of unsaturated bonds as well as the location of these bonds on the hydrocarbon chains are crucial for a lipid’s T_m_ and its overall thermotropic behavior [31,32,36]. As far as copolymers are concerned, all of them were used in lipid to polymer weight ratios of 9:1, 7:3, and 5:5. In fact, for these ratios it was observed that there is a linear correlation between the %weight of copolymer and enthalpy values. All calorimetric results are presented in Tables S6–S9.

**Figure 4:**
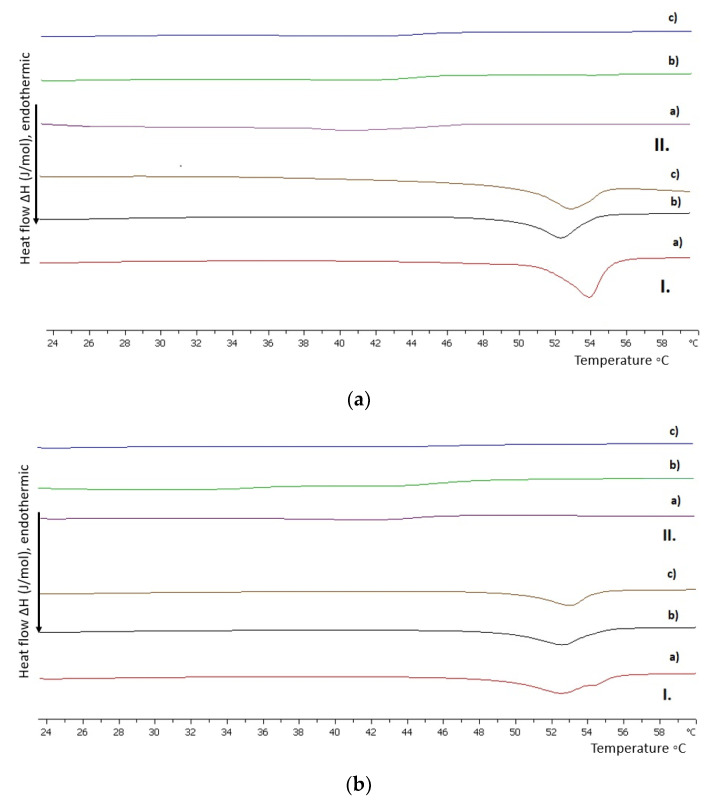
DSC thermograms during heating of hydrated (**a**) DSPC:DOPC:P(OEGMA_950_-DIPAEMA)-1 and (**b**) DSPC:DOPC:P(OEGMA_950_-DIPAEMA)-2 hybrid bilayers in different DSPC:DOPC weight ratios, (**I**) 9:1 and (**II**) 4:6, as well as different lipid to polymer weight ratios: (a) 9:1, (b) 7:3, and (c) 5:5.

Generally, the incorporation of and increase in DOPC into the membrane lead to more fluid bilayers for all the hybrid systems investigated (Figure 4). The pretransition is absent in all cases due to modifications in the mobility of the polar head group region [38,46], while the peak width at half height is higher and T_m_ is decreasing, as is the enthalpy. The double bonds of DOPC in the center of the aliphatic chains favor the decrease in van der Waals interactions in the hydrophobic interior, permitting conformational freedom [32].

As far as the DSPC:DOPC 9:1 hybrid system is concerned, there are a few points that should be highlighted. Even though there is no trend in calorimetric parameters by increasing copolymer amount, enthalpy values lead to an interesting conclusion. The copolymer P(OEGMA_950_-DIPAEMA)-1 seems to occupy equivalent space in the bilayer as the respective DSPC hybrid systems despite the existence of another lipid which influences the hydrophobic interior. This observation strengthens our opinion about the copolymer’s location in the bilayer. Additionally, the utilization of P(OEGMA_950_-DIPAEMA)-2 in the DSPC:DOPC 9:1 bilayer shows a wide shoulder, as the heating profile illustrates in Figure 4, indicating disassociation and lateral phase separation [41,42].

For the hybrid systems with the lipid part at a 4:6 weight ratio there is a fluidization effect in all cases. It is well known that polymers stabilize lipidic systems [47,48]. In this case, this is a result of the long OEGMA chains and is driven by hydrogen bonding and steric effects [37]. However, fluidization of the membrane for DSPC:DOPC 4:6 hybrid systems is inevitable due to the predominance of DOPC and its tendency for chain disorder due to “kinks” in the tails [32,49]. To the best of the authors’ opinion, the random copolymer design was unable to significantly control the bilayer disorder or orientation variability, which is what causes the fluidization effect [38,41,50].

Comparing the equivalent systems of P(OEGMA_950_-DIPAEMA)-1 and -2, the following conclusions can be made. The area of the respective endothermic peaks for copolymer P(OEGMA_950_-DIPAEMA)-2, the cooperativity, and the temperature of the main transition are decreased a bit. We could assume that the increase in the partially deprotonated and bulky DIPAEMA as well as the unsaturated hydrocarbon chains of DOPC contribute to a greater distance among the lipids and a more fluid bilayer with more configurational option results [49]. The cooling processes are reversible, with slight hysteresis in all cases and higher enthalpy values, as can be observed in the Supplementary Material (Tables S7 and S8, respectively).

### 3.2. Physicochemical Evaluation of Lipid/Polymer Hybrid Nanosystems

#### 3.2.1. The Influence of Chemical Composition and Lipid to Copolymer Ratio

Based on the above preliminary results, hybrid nanoparticles were formed utilizing as the lipidic part DSPC or DSPC:DOPC 9:1 to examine how the different biophysical properties influence their physicochemical characteristics and biocompatibility. The 4:6 weight ratio of the lipid mixture was eliminated due to a pronounced fluidization effect. The utilization of the copolymer P(OEGMA_950_-DIPAEMA)-1 confers on hybrid systems an R_h_ equal to or less than 100 nm in water and homogenous in general populations according to PDI values, except for DSPC:DOPC:P(OEGMA_950_-DIPAEMA)-1 9:1 and 5:5 which show slight heterogeneity in size distribution (Table 2) [51,52]. However, the lipid composition is an important parameter that affects size [53]. DSPC:DOPC hybrid systems generally have similar dimensions to respective DSPC nanoparticles, with the exception of the 9:1 lipid to polymer ratio. Scattering intensity, which is correlated with the mass of the nanostructure [52,54], is not proportional to copolymer ratio increase. Although the increase in copolymer content at a weight ratio of 7:3 leads to an enormous increase in intensity, regardless of the lipid composition, more addition of copolymer (50% weight) corresponded to an evident reduction in the I value. This is partially explained by a proportional alteration of the hydrodynamic radius [52]. More importantly, this finding demonstrates the impact of the lipid to polymer ratio, and it might be a result of random copolymer architecture and composition. Namely, the increase in entry and exit points in the bilayer in combination with the bulky DIPAEMA, which causes steric hindrance, may provoke rearrangement of the structure to reduce the surface energy due to disconformation and different intra-molecular interactions [55]. This explanation is further supported by fluctuations in the rest of the physicochemical characteristics that are discussed below.

According to SLS measurements, the R_g_/R_h_ ratio indicates that the morphology of all the systems is estimated as a rather loose conformation in the range of a sphere with thin walls to a random coil [56]. The micropolarity of the systems is between 1.62 to 1.78, indicating a highly hydrophilic environment [57]. Probably, the highly polar microenvironment of all hybrid nanostructures that pyrene perceived is owed to the chemical composition and the morphology that permits the existence of the solvent or/and some hydrophilic OEGMA chains in the interior.

As illustrated in Table 2, the increase in the partially deprotonated and bulky DIPAEMA in the random copolymer P(OEGMA_950_-DIPAEMA)-2 led to variability in size for all the systems, with R_h_ ranging between 16 to 124 nm and in some cases the existence of a second population. Therefore, the polydispersity index suggests slight heterogeneity for the systems, with values ranging from 0.42 to 0.46, whereas there are some homogenous populations for the DSPC:P(OEGMA_950_-DIPAEMA)-2 5:5 and DSPC:DOPC:P(OEGMA_950_-DIPAEMA)-2 9:1 and 7:3 systems. Moreover, the addition of DOPC in the hybrid systems for a constant lipid to polymer ratio results in larger structures than neat DSPC. This is not in accordance with liposome performance [49,53]. Conceivably, it is due to the hybrid nature of the nanostructures [55]. Some systems exhibit a second population with a quite small size in all cases (16–17 nm). It is our hypothesis that the small-sized population could correspond to neat copolymer self-assemblies, but it should be further investigated by other techniques. In fact, it has been mentioned before the single-chain aggregates of random copolymers driven by hydrophobic interactions [58]. Intensity differs for every system and there are no specific trends.

The heterogeneity of the systems did not favor the measurement of R_g_/R_h_ for most of the systems apart from DSPC:DOPC:P(OEGMA_950_-DIPAEMA)-2 9:1, which resembles a configuration of a random coil in accordance with most P(OEGMA_950_-DIPAEMA)-1 hybrid systems. The I_1_/I_3_ values point out a polar microenvironment with no specific patterns, highlighting the unique properties of every hybrid structure. However, the addition of DOPC slightly diminishes micropolarity, while the increase in %DIPAEMA in copolymer 2 leads to reduced values compared to the respective systems of P(OEGMA_950_-DIPAEMA)-1. This could be attributed to increased surface hydrophobicity as a result of increased hydration and fluidity of the membrane and thus a possible different internal arrangement of the hybrid nanoparticles [59].

Physicochemical characteristics (R_h_, I) of hybrid systems comprised of P(OEGMA_950_-DIPAEMA)-1 present higher values compared to respective P(OEGMA_950_-DIPAEMA)-2 systems, while copolymer 2 systems show the co-existence of two populations occasionally. In both cases the particle size is acceptable for systemic administration and sustained release [7,29,60,61]. The greater amount of OEGMA chains of copolymer 1 induces a denser surface on the exterior region, likely equivalent to a brush regime, and thus the observation of larger structures for P(OEGMA_950_-DIPAEMA)-1 [62,63]. Furthermore, the increase in % PDIPAEMA of the random P(OEGMA_950_-DIPAEMA)-2 copolymer and therefore the different hydrophilic to hydrophobic ratio of the polymer advocate different self-assemblies of the nanostructures, which are depicted in their less toxic behavior, as is described in the nanotoxicity section. However, the hydrophobic to hydrophilic ratio cannot be confronted simply as a factor affecting the dimensionless packing parameter, such as in the case of block copolymer structures. It requires a more systemic approach, since in these copolymers the hydrophobic and hydrophilic segments are spread randomly along the polymer chain and it might be referring to a balance between hydrophilic and hydrophobic segments and interactions [33,58,64]. In this manner, the fact that each hybrid system exhibits an exceptional physicochemical behavior is rational and is interpreted via different intra- and inter-molecular interactions, entropic phenomena, and unique membrane distribution due to the different nature of the individual biomaterials and the random architecture of the copolymer [33,51,55,58,62,65]. Generally, no specific patterns are observed in the results of Section 3.2.2 either. In fact, the unique behavior of the different hybrid systems in the environmental conditions could resemble the schizophrenic character of block copolymers [66,67]. However, in this case it is the result of different interactions between the molecules leading to different self-assemblies, as aforementioned, and not the responsiveness of both comonomers.

#### 3.2.2. The Impact of Stimuli-Responsive Copolymers on the Physicochemical Properties of Hybrid Nanostructures

**Figure 5:**
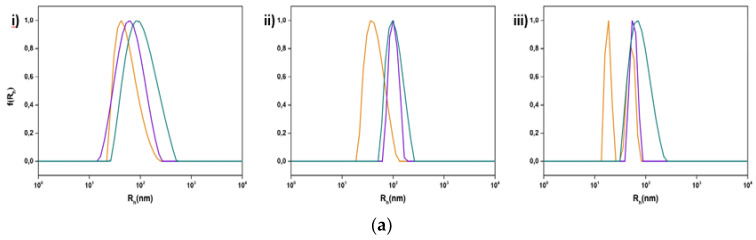

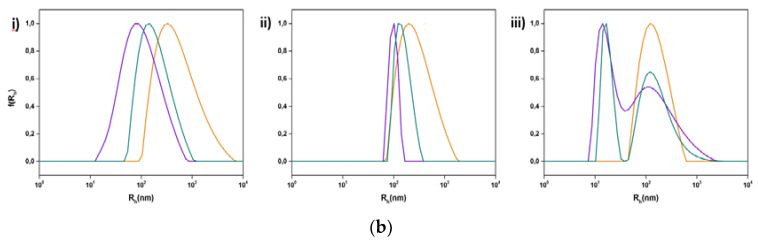
Size distributions from DLS for the hybrid systems incorporating (**a**) DSPC:P(OEGMA_950_-DIPAEMA)-1 and (**b**) DSPC:DOPC:P(OEGMA_950_-DIPAEMA)-1 in three different lipid to polymer weight ratios, (**i**) 9:1, (**ii**) 7:3, and (**iii**) 5:5, and three different pH media: HCl 0.1 N (pH 1.2) (orange color), water for injection (pH 5.5) (purple color), and PBS (pH 7.4) (green color).

**Figure 6:**
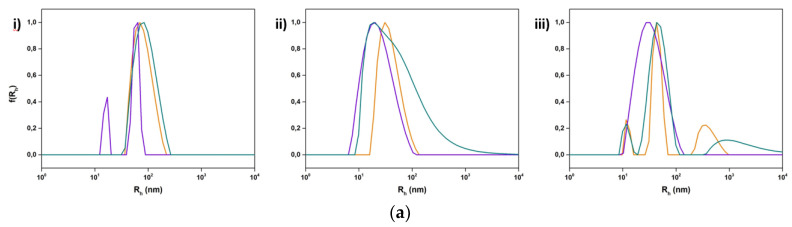

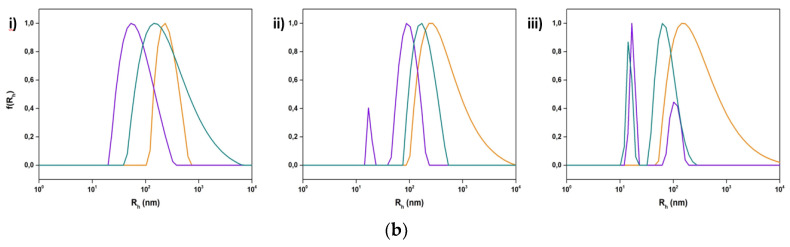
Size distributions from DLS for the hybrid systems incorporating (**a**) DSPC:P(OEGMA_950_-DIPAEMA)-2 and (**b**) DSPC:DOPC:P(OEGMA_950_-DIPAEMA)-2 in three different lipid to polymer weight ratios, (**i**) 9:1, (**ii**) 7:3, and (**iii**) 5:5, and three different pH media: HCl 0.1 N (pH 1.2) (orange color), water for injection (pH 5.5) (purple color), and PBS (pH 7.4) (green color).

Concerning pH modifications, the incorporation of P(OEGMA_950_-DIPAEMA) copolymer 1 or 2 in the nanosystems accredits to different physicochemical behaviors, which is reported in Figure 5 and Figure 6 as well as in Tables S10 and S11. For hybrid systems of P(OEGMA_950_-DIPAEMA)-1, different patterns are observed at pH 1.2 regarding lipid composition, particularly smaller hybrid nanostructures of DSPC and larger of DSPC:DOPC. In general, the mass of colloidal systems based on intensity values shows fluctuations in a similar manner to particle size [52]. The increase in the dimensions of DSPC:DOPC hybrid systems could be linked to a swollen structure due to the protonated amino group carrying segments that are fully stretched, in accordance with PDIPAEMA behavior in other nanoparticles [68]. The opposite results of DSPC hybrid systems evince different interactions according to extended DLVO theory [51]. Moreover, we should keep in mind that the membrane mechanics, which affect morphology, are associated with the packing order of the bilayer [69]. In correlation to DSC experiments in HCl 0.1 N (see Section 3.1.2), a more rigid DSPC hybrid membrane is expected due to the significant increase in T_m_ [70]. By increasing the copolymer to lipid ratio the size is reduced for DSPC:DOPC systems, while it remains almost unaffected for DSPC ones, with the exception of DSPC:P(OEGMA_950_-DIPAEMA)-1 5:5 consisting of two populations in an equal amount. This decrease in the R_h_ might be due to more entry and exit points of the highly polar groups of the random copolymer in the lipid bilayer, leading to different interactions, increased surface curvature, and thus smaller size [55]. In addition, DSPC:P(OEGMA_950_-DIPAEMA)-1 5:5 results could be supported by the aforementioned DSC study due to new metastable phases in acidic pH, which may correspond to two different conformations that co-exist. Presumably, the combination of cationic amino groups and OEGMA segments with a phosphatidylcholine head group provokes electrostatic interactions and dehydration of hydrophilic chains, resulting in modifications in the interfacial region and thus OEGMA chain entanglement and a phase separation [71,72].

Furthermore, in PBS the nanoplatforms integrating copolymer 1 increase their size in comparison to water, mainly due to steric effects and hydration forces [51,62]. Different patterns regarding lipid composition are also applied. Namely, for DSPC hybrid systems the gradual increase in pH is accompanied by a concurrent increase in the hydrodynamic radius. The gamut of intensity values depending on the lipid to polymer ratio possibly reflects the contribution of random copolymer architecture. On the contrary, the change from an acidic to an almost neutral pH, slightly alkaline, was illustrated by smaller-sized DSPC:DOPC configurations with larger mass. The more compact aggregates due to pH change have been mentioned before for PDIPAEMA nanostructures [68]. Despite the different physicochemical results, in all cases the decrease in hydrophilicity of the copolymer due to partial deprotonation of the amine group as well as hydration repulsions and osmotic phenomena due to the existence of PBS ions are the driving forces for the rearrangement of the molecules to different structures [51,73].

The predominance of pH-responsive PDIPAEMA in the hybrid nanostructures by incorporating P(OEGMA_950_-DIPAEMA)-2 indicates major modulations on the system properties. In principle, the influence of either a fully protonated or partially deprotonated amine group is prominent, because size is increased compared to the aqueous environment for both dispersion media and systems tend toward polydispersity (PDI values from 0.4 to 0.5). For the lipid to polymer ratio 9:1 R_h_ is very similar in acidic and PBS media, highlighting the small impact on the structure due to the low amount of copolymer. Scattered light intensity, which is a sensitive parameter for the mass of the nanoparticle, differs for every hybrid system, with no specific trends to be reported. This outcome demonstrates the exceptional properties of each system owing to random copolymer topology, as has already been mentioned, and is strengthened by observing the DSPC hybrid system of the 50% copolymer. In both pH media aggregates are formed, and especially at an acidic pH nanoassembly morphology homogeneity is completely ruined, as three different-sized populations co-exist. The instability of hybrid nanoparticles with a low proportion of PEG chains in a PBS environment has been reported in the literature as well [74].

**Figure 7:**
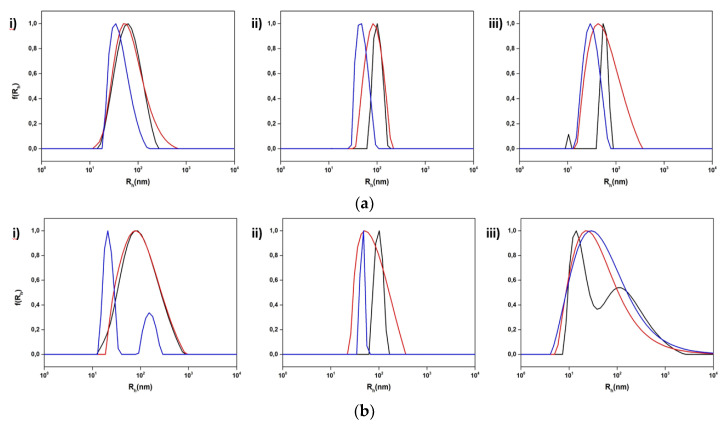
Size distributions from DLS for the hybrid systems incorporating (**a**) DSPC:P(OEGMA_950_-co-DIPAEMA)-1 and (**b**) DSPC:DOPC:P(OEGMA_950_-co-DIPAEMA)-1 in three different lipid to polymer weight ratios, (**i**) 9:1, (**ii**) 7:3, and (**iii**) 5:5, and three different temperatures: 25 °C (black color), 37 °C (red color), and 60 °C (blue color).

Referring to temperature changes, at 37 °C the size of the P(OEGMA_950_-DIPAEMA)-1 hybrid systems remained more or less unaffected, with the exception of the lipid to polymer ratio 7:3 for which the size decreased (Figure 7 and Table S12). By increasing the temperature above the main phase transition temperature of the bilayer, the dimensions as well as the mass of the colloidal system are mainly decreased. This could involve the liquid crystalline state of the lipids leading to partial disorganization of the nanoassemblies [73].

**Figure 8:**
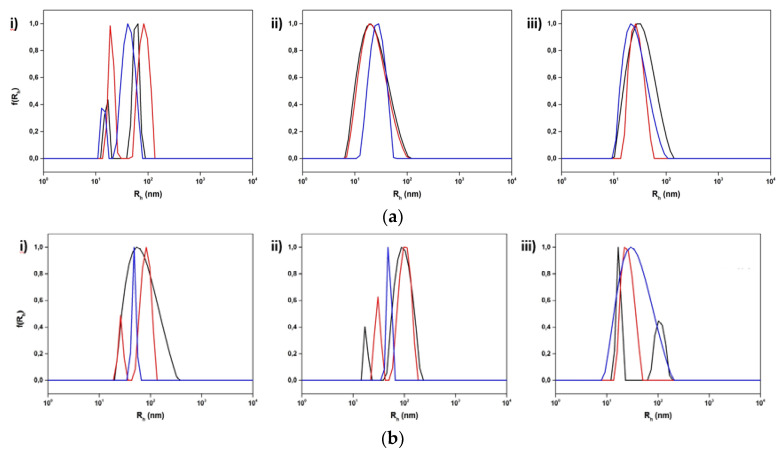
Size distributions from DLS for the hybrid systems incorporating (**a**) DSPC:P(OEGMA_950_-co-DIPAEMA)-2 and (**b**) DSPC:DOPC:P(OEGMA_950_-co-DIPAEMA)-2 in three different lipid to polymer weight ratios, (**i**) 9:1, (**ii**) 7:3, and (**iii**) 5:5, and three different temperatures: 25 °C (black color), 37 °C (red color), and 60 °C (blue color).

The presence of P(OEGMA_950_-DIPAEMA)-2 caused more intense modifications in the physicochemical characteristics of the systems at 37 °C, owing perhaps to the increased proportion of the thermoresponsive DIPAEMA segment (Figure 8 and Table S13). There are no specific patterns, however, neither for R_h_ nor for the intensity, probably due to the random architecture. At 60 °C the size is reduced or maintained, and the intensity is mainly decreased in a similar manner to P(OEGMA_950_-DIPAEMA)-1 copolymer nanoplatforms.

### 3.3. In Vitro Toxicity Studies

**Figure 9:**
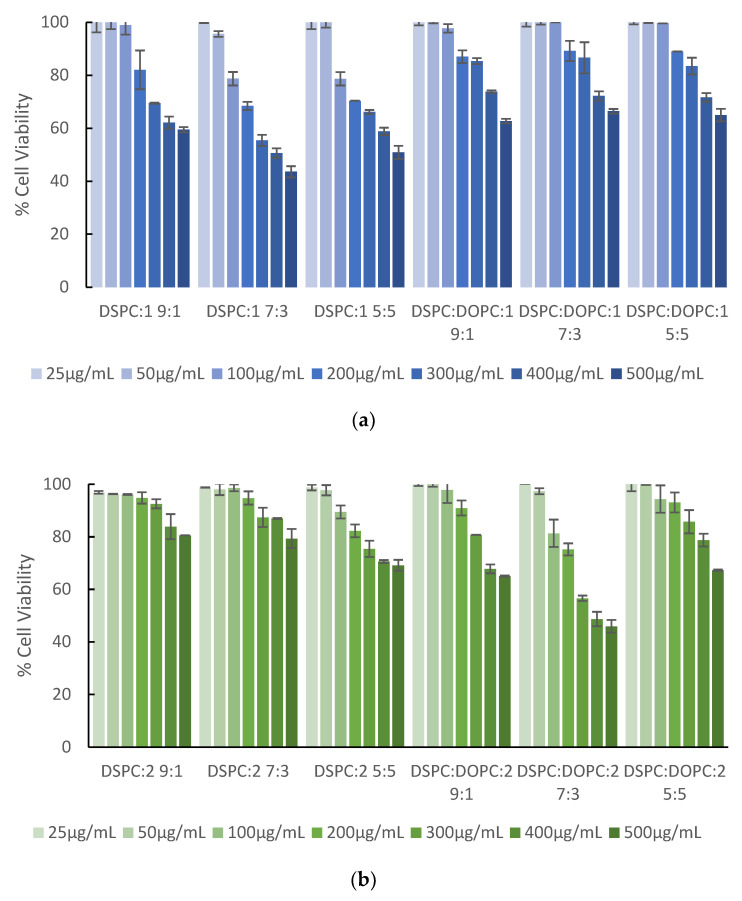
Cell viability vs. different concentrations of DSPC and DSPC:DOPC 9:1 hybrid systems with incorporated copolymer (**a**) P(OEGMA_950_-co-DIPAEMA)-1 or (**b**) P(OEGMA_950_-co-DIPAEMA)-2 at different lipid to polymer weight ratios. The error bars represent the standard deviation.

The main purpose of the study was to investigate whether lipid/copolymer nanostructures are biocompatible to be used as potential drug carriers. Furthermore, we attempted to elucidate the correlation between their characteristics and toxic behavior so as to examine which parameters affect their biocompatibility the most. All the systems were studied for their biocompatibility via the MTS protocol. The % cell viability of HEK293 cell lines was correlated to different concentrations of the hybrid nanoplatforms and the results are shown in Figure 9. All hybrid systems display a dose-dependent cytotoxicity and are biocompatible for at least a 100 μg/mL concentration. For most of the systems the cell viability is above 60% in all tested concentrations. The maximum concentration of the copolymer that was examined for its toxicity based on the hybrid system weight ratio is 250 μg/mL for 5:5 hybrid systems.

Among the hybrid systems of DSPC:P(OEGMA_950_-DIPAEMA)-1, the best toxicological profile is related to the DSPC:P(OEGMA_950_-DIPAEMA)-1 9:1 system, since cell viability is more than 80% until 200 μg/mL. Interestingly, the presence of DOPC ameliorates the toxicological behavior. In particular, viability was above 80% for the high concentration of 300 μg/mL and did not drop under 60% for any of the examined concentrations. The main reason seems to be a different morphology for these systems, as physicochemical measurements imply. The effect of shape and morphology in the observed nanotoxicity is well established [39,75,76,77]. Considering the thermodynamic properties, we speculate that this significant change in cytotoxicity with DOPC addition is related not only to the different configurations of the nanostructure but also to the fluidization of the bilayer.

Regarding DSPC:P(OEGMA_950_-DIPAEMA)-2 hybrid systems, there is a lipid-to-polymer-dependent toxicity. For all the examined concentrations, the viable cells are more than 80% for the 9:1 and 7:3 lipid to polymer ratios and more than 70% for 5:5. With the incorporation of DOPC in the hybrid systems there is no more lipid-to-polymer-dependent pattern, with the most cytotoxic behavior belonging to the 7:3 hybrid system. Moreover, the addition of DOPC increases the cytotoxicity for the lipid to polymer ratios 9:1 and 7:3. However, the cell viability is still high and mostly over 60%. The 5:5 system is sufficiently biocompatible; its cytotoxic effect does not exceed 30% at the highest examined concentration level (500 μg/mL) corresponding to a maximum copolymer concentration of 250 μg/mL. Even though we could not possibly be sure about the morphology and the constitution of the hybrid nanoplatforms yet, we could hypothesize that the existence of a second small-sized population is the reason for a better toxicological profile of this system. It is noteworthy that the systems exhibiting the greatest cell viability are those with one lipid component, in contrast to the copolymer 1 results.

Generally, the nanostructures utilizing P(OEGMA_950_-DIPAEMA)-2 over 1 are more biocompatible, apart from some exceptions. It seems that different hydrophobic to hydrophilic ratios play a crucial role in the cytotoxic profile, probably due to different interactions inducing alterations in self-assembly and shape of the nanoparticles, as aforementioned. The elucidation of the parameters affecting nanotoxicity of these systems requires further investigation via imaging techniques to come to certain conclusions.

## 4. Conclusions

For the first time, hybrid nanostructures composed of DSPC, DOPC, and the linear random copolymer P(OEGMA_950_-co-DIPAEMA) were successfully prepared and examined for their physicochemical and toxicological properties. Additionally, their thermotropic characteristics as hybrid lipid membrane components were studied and interpreted. The presence of DOPC and the consequent increased fluidity altered to a great extent the biophysical properties of the hybrid bilayers compared to the DSPC hybrid systems. From a copolymer perspective, it seems that lipid to polymer ratio as well as the hydrophilic to hydrophobic ratio of the copolymer and the random architecture are crucial parameters for the incorporation of the polymers into the bilayer. The different interactions between the biomaterials lead to different self-assemblies and morphologies. The physicochemical results confirm the above, pointing out the importance of the random architecture, since its hybrid nanostructures exhibits exclusive features. Albeit the exceptional behavior of each nanoplatform, all systems show pH- and thermoresponsive characteristics, which we assume would influence the drug release profile. Temperature and pH sensitivities are mainly the result of copolymer incorporation and especially of DIPAEMA’s ability to change at a molecular level, altering from hydrophilic to hydrophobic and vice versa depending on environmental conditions. In this regard, DSPC:P(OEGMA_950_-co-DIPAEMA) or DSPC:DOPC:P(OEGMA_950_-co-DIPAEMA) nanostructures could be promising nanoplatforms for modified release. In fact, the unique characteristics in different environmental conditions give the opportunity for each hybrid system to be utilized for a different purpose in novel therapy schemes. For instance, they could be used in cancer therapy by exploiting differentiations in tumor pH or in combination with hyperthermia for targeted drug release. They might also be useful in synergistic treatment or in the theragnostic field. Moreover, due to their size, which is not exceeding 200 nm in most cases, they could be utilized by different routes of administration, including intravenous or intramuscular. Finally, the in vitro toxicity investigation indicates that most of the hybrid nanostructures are non-toxic at low concentrations, while the cytotoxicity seems to be affected by specific parameters, including hydrophilic/hydrophobic balance and hybrid nanostructure morphology.

## Figures and Tables

**Table 1:** Chemical properties of copolymers used in this study.

Statistical (Random) Copolymers			
P(OEGMA ¹ _950_-co-DIPAEMA ²)		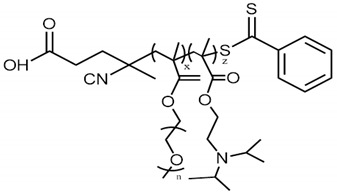	
	M_w_ ^4^ (×10^4^) (g/mol)	M_w_/M_n_ ^4^	%PDIPAEMA ^3^
Copolymer 1	1.10	1.16	37
Copolymer 2	1.24	1.13	70

^1^ OEGMA: oligo (ethylene glycol) methyl ether methacrylate. ^2^ DIPAEMA: 2-[diisopropylamino] ethyl methacrylate. ^3^ Determined by ^1^H-NMR in CDCl_3_. ^4^ Determined by SEC in THF at 25 °C.

**Table 2:** Physicochemical properties of hybrid systems incorporating P(OEGMA_950_-DIPAEMA) copolymers 1 and 2 at 25 °C and in water for injection dispersion medium.

Sample	Weight Ratio	Ι (kHz)	R_h_ (nm)	PDI	R_g_/R_h_	I_1_/I_3_
DSPC:1	9:1	4210	62	0.31	1.4	1.65
DSPC:1	7:3	21,645	103	0.20	1.3	1.78
DSPC:1	5:5	3023	58	0.31	1.4	1.68
DSPC:DOPC:1	9:1	4230	95	0.40	1.5	1.62
DSPC:DOPC:1	7:3	16,419	99	0.27	1.1	1.63
DSPC:DOPC:1	5:5	192	57	0.52	1.2	1.69
DSPC:2	9:1	1087	58 (76% wP) ^1^	0.42	-	1.68
DSPC:2	7:3	413	23	0.46	-	1.67
DSPC:2	5:5	499	33	0.27	-	1.58
DSPC:DOPC:2	9:1	3970	69	0.28	1.4	1.61
DSPC:DOPC:2	7:3	4730	94 (92% wP) ^2^	0.33	-	1.50
DSPC:DOPC:2	5:5	493	108 (44% wP) ^3^	0.46	-	1.60

^1^ Co-existence of another size population at 16 nm (23% wP), ^2^ co-existence of another population at 17 nm (8% wP), and ^3^ co-existence of another population at 18 nm (55% wP).

## Data Availability

Not applicable.

## Supplementary Materials

The following supporting information can be downloaded at: https://www.mdpi.com/article/10.3390/polym15091989/s1, Synthesis of P(OEGMA_950_-co-DIPAEMA) linear copolymers; Scheme S1: Synthetic route for the P(OEGMA-co-DIPAEMA) statistical copolymers; Figure S1: SEC chromatograms of the P(OEGMA-co-DIPAEMA) statistical copolymers, (i) copolymer 1 and (ii) copolymer 2, before (black line) and after (red line) dialysis method; Figure S2: Typical ^1^H-NMR spectrum of P(OEGMA-co-DIPAEMA)-1 statistical copolymer; Table S1: Chemical properties of lipids used in this study; Table S2: Calorimetric parameters of DSPC:P(OEGMA_950_-co-DIPAEMA) hybrid bilayers during heating; Table S3: Calorimetric parameters of DSPC:P(OEGMA_950_-co-DIPAEMA) hybrid bilayers during cooling; Table S4: Calorimetric parameters of DSPC:P(OEGMA_950_-co-DIPAEMA) hybrid bilayers during heating in dilution medium HCl 0.1 N (pH 1.2); Table S5: Calorimetric parameters of DSPC:P(OEGMA_950_-co-DIPAEMA) hybrid bilayers during cooling in dilution medium HCl 0.1 N (pH 1.2); Table S6: Calorimetric parameters of DSPC:DOPC:P(OEGMA_950_-co-DIPAEMA)-1 hybrid bilayers during heating; Table S7: Calorimetric parameters of DSPC: DOPC:P(OEGMA_950_-co-DIPAEMA)-1 hybrid bilayers during cooling; Table S8: Calorimetric parameters of DSPC:DOPC:P(OEGMA_950_-co-DIPAEMA)-2 hybrid bilayers during heating; Table S9: Calorimetric parameters of DSPC:DOPC:P(OEGMA_950_-co-DIPAEMA)-2 hybrid bilayers during cooling; Table S10: Physicochemical properties of hybrid systems incorporating copolymer P(OEGMA_950_-DIPAEMA)-1 at 25 °C and in different pH media; Table S11: Physicochemical properties of hybrid systems incorporating copolymer P(OEGMA_950_-DIPAEMA)-2 at 25 °C and in different pH media; Table S12: Physicochemical properties of hybrid systems incorporating copolymer P(OEGMA_950_-DIPAEMA)-1 at different temperatures and in water for injection dispersion medium (WFI); Table S13: Physicochemical properties of hybrid systems incorporating copolymer P(OEGMA_950_-DIPAEMA)-2 at different temperatures and in water for injection dispersion medium (WFI). References [78,79,80,81,82,83] are cited in the supplementary materials.
