# Supplementary material for: Development of Hybrid DSPC:DOPC:P(OEGMA950-DIPAEMA) Nanostructures: The Random Architecture of Polymeric Guest as a Key Design Parameter
Source: Polymers (Basel). 2023 Apr 22;15(9):1989. doi: 10.3390/polym15091989 (PMC10181429; doi:10.3390/polym15091989)
Supplement: Supplementary file 1 [file polymers-15-01989-s001.zip › polymers-2348443-supplementary.pdf]

Supplementary Material

Development of Hybrid DSPC:DOPC:P(OEGMA$_{950}$-DIPAEMA) Nanostructures: The Random Architecture of Polymeric Guest as a Key Design Parameter

Efstathia Triantafyllopoulou [1], Dimitrios Selianitis [2], Natassa Pippa [1], Maria Gazouli [3], Georgia Valsami [1], and Stergios Pispas [2,*]

Synthesis of P(OEGMA$_{950}$-co-DIPAEMA) linear copolymers

P(OEGMA$_{950}$-co-DIPAEMA) copolymers were synthesized via RAFT polymerization utilizing 4-cyano-4-(phenylcarbonothioylthio) pentanoic acid (CPAD) as the chain transfer agent (CTA) as well as the azobis(isobutyronitrile) (AIBN) as the radical initiator. The solvent of the chemical reaction was the 1,4-dioxane. A typical example of the synthesis is described below: OEGMA$_{950}$ (0.7g, 0.73mmol), DIPAEMA (0.3g, 1.4 mol) CPAD (0.095g, 0.34mmol), AIBN (0.011g, 0.067mmol) 1,4-dioxane (3.5mL) were added to an one-necked, round bottom flask containing a magnetic stir bar and fitted with a rubber septum. The mixed solution was degassed by nitrogen flow for 20min and then the flask placed at 70oC in a thermostated oil bath for 24h. Afterwards, the chemical reaction was placed at -20oC for 15min and then exposed to air. Subsequently, the product of the reaction was precipitated in a large excess of n-hexane for removal of the unreacted monomers. Last, the copolymer was placed in a vacuum oven for 48h for drying.

Two P(OEGMA$_{950}$-co-DIPAEMA) linear statistical (random) copolymers were synthesized by RAFT methodology. The selection of CPAD as the CTA for the polymerization was made because it is an effective RAFT agent for methacrylic monomers [78]. Schematic illustration for the synthesis of P(OEGMA$_{950}$-co-DIPAEMA) copolymer is presented in Scheme S1. Typical chromatograms of P(OEGMA$_{950}$-co-DIPAEMA) copolymers are illustrated in Figure S1. The molecular weights as well as the molecular weight distributions of the synthesized copolymers in the present work were determined by size exclusion chromatography (SEC). Analysis of the chromatograms presented a relatively good control of the copolymer molecular weight. However, it was necessary to use the dialysis method on the copolymer to remove the unreacted monomers (Figure S1). According to the chromatogram, the molecular weight distributions were low and comparable to the theoretical background of the RAFT polymerization method [79].

Chemical identification of the synthesized copolymers as well as their chemical composition were determined by ^{1}H-NMR spectroscopy. Characteristic spectra are presented in Figure S2. Based on the indicative 1H-NMR spectra, differences between

the peak intensity are observed. This is due to the different monomeric units composing the statistical (random) copolymers. In all cases the most characteristic peaks were used. In particular, for OEGMA the -CH2 hydrogens of the side group of oligoethylene glycol were selected (peak g, 3.63ppm) [80] and the (CH-N)2 hydrogens of the tertiary carbons of the two isopropyl groups (peak e, 2.99ppm) [81].

Scheme S1. Synthetic route for the P(OEGMA-co-DIPAEMA) statistical copolymers.

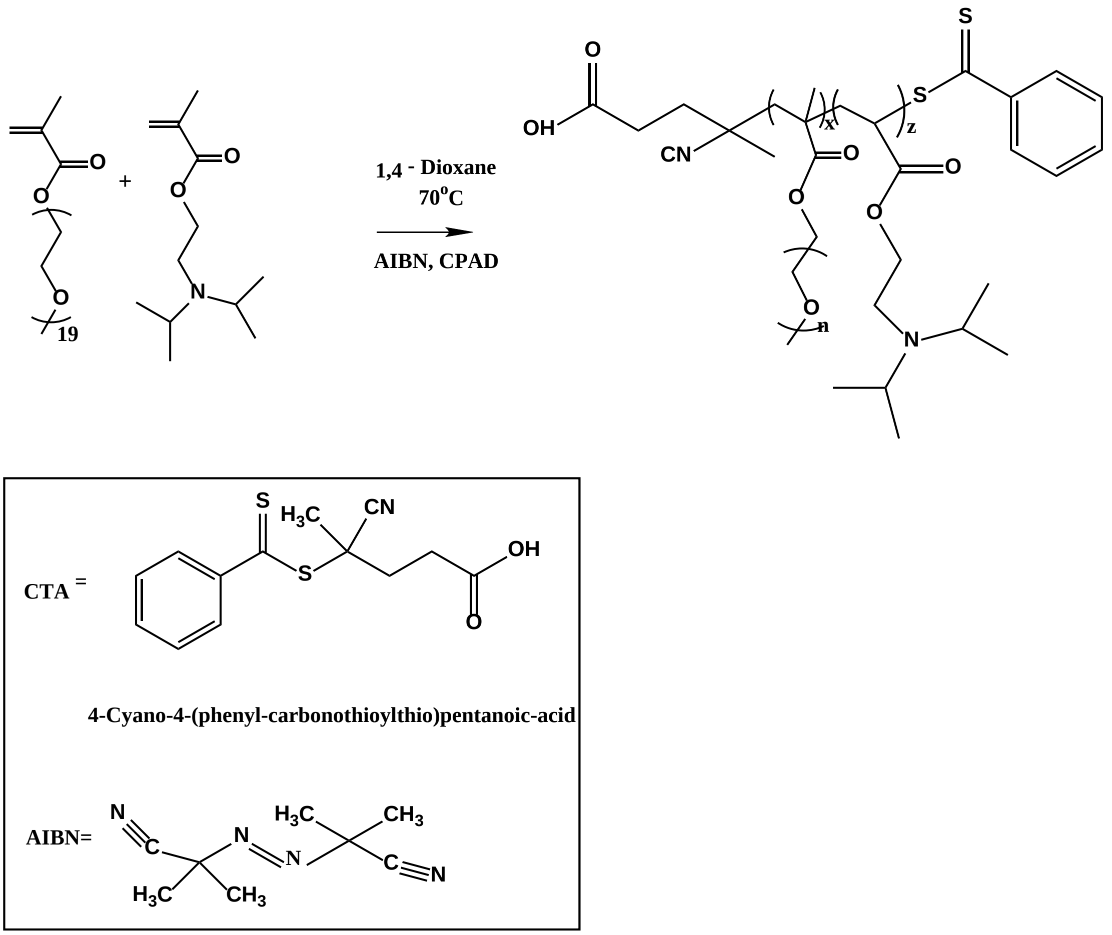

Figure S1. SEC chromatograms of the P(OEGMA-co-DIPAEMA) statistical copolymers: i) copolymer 1 and ii) copolymer 2, before (black line) and after (red line) dialysis method.

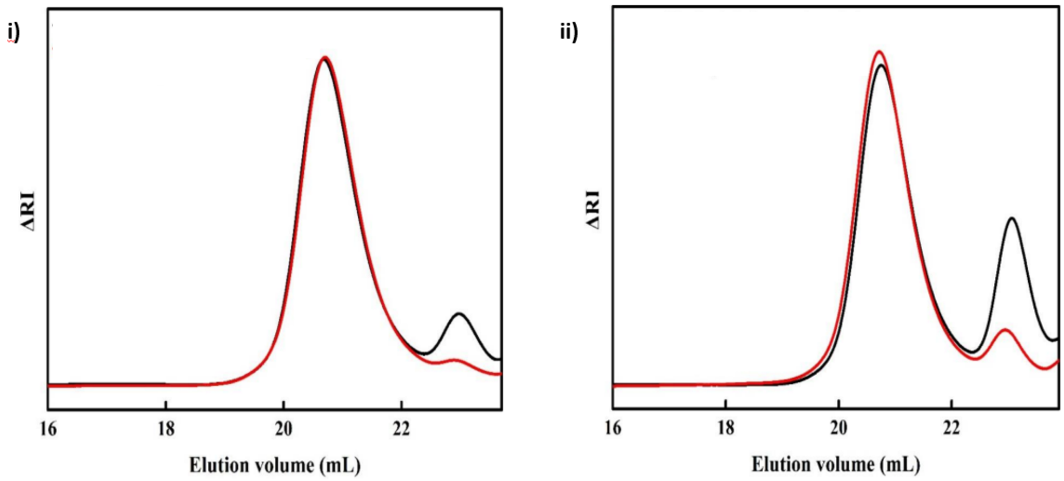

Figure S2. Typical ^{1}H-NMR spectrum of P(OEGMA-co-DIPAEMA)-1 statistical copolymer.

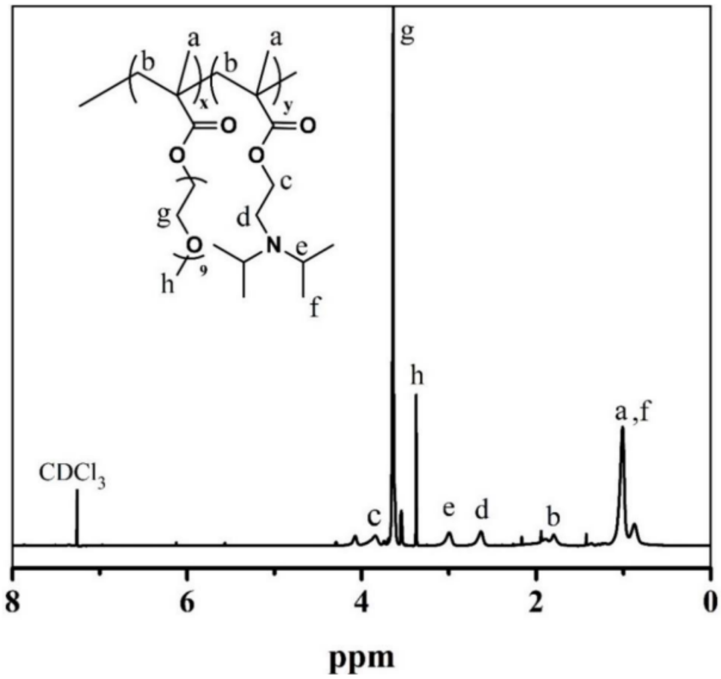

Table S1. Chemical properties of lipids used in this study.

Lipids					
**Lipid**	**Chemical Name**	**Molecular Weight**	**T_m (°C)**	**Acyl chains[1]**	**Skeletal Formula**
**DSPC**	1,2-dioctadecanoyl-sn-glycero-3-phosphocholine	789.625	55	18:0/18:0	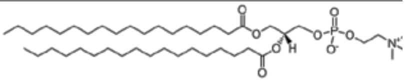
**DOPC**	1,2-dioleoyl-sn-glycero-3-phosphocholine	785.593	-17	18:1(9Z)/ 18:1(9Z)	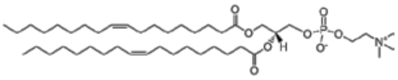

[1]Number of C: unsaturated bonds

Adapted from [82, 83].

Table S2. Calorimetric parameters of DSPC:P(OEGMA$_{950}$-co-DIPAEMA) hybrid bilayers during heating.

Weight ratio	$T_{onset,m}$ (°C)[1]	T_m (°C)[2]	$\Delta T_{½,m}$ (°C)[3]	ΔH_m (J/mol)[4]	$T_{onset,s}$ (°C)	T_s (°C)	$\Delta T_{½,s}$ (°C)	ΔH_s (J/mol)
**DSPC:P(OEGMA$_{950}$-co-DIPAEMA)-1**								
**9:1**	54.2	55.2	1.16	366	48.6	50.8	2.60	28
**8:2**	54.0	55.1	1.23	379	47.8	50.4	2.91	24
**7:3**	53.9	54.9	1.19	275	-	-	-	-
**6:4**	53.8	54.8	1.27	307	-	-	-	-
**5:5**	53.7	54.9	1.36	223	-	-	-	-
**DSPC:P(OEGMA$_{950}$-co-DIPAEMA)-2**								
**9:1**	53.9	55.0	1.12	403	48.1	50.3	2.23	12
**8:2**	53.3	54.8	1.36	404	-	-	-	-
**7:3**	53.2	54.7	1.35	300	-	-	-	-
**6:4**	54.0	55.1	1.27	286	-	-	-	-
**5:5**	52.9	54.8	1.62	168	-	-	-	-

[1] Tonset : temperature at which the thermal event starts
[2] T : temperature at which heat capacity (ΔCp) at constant pressure is maximum
[3] ΔT½ : half width at half peak height of the transition
[4] ΔH : transition enthalpy normalized per mol of lipid system
m : main transition, **s** : secondary transition

Table S3. Calorimetric parameters of DSPC:P(OEGMA$_{950}$-co-DIPAEMA hybrid bilayers during cooling.

Weight ratio	$T_{onset,m}$ (°C)[1]	T_m (°C)[2]	$\Delta T_{½,m}$ (°C)[3]	ΔH_m (J/mol)[4]	$T_{onset,s}$ (°C)	T_s (°C)	$\Delta T_{½,s}$ (°C)	ΔH_s (J/mol)
DSPC:P(OEGMA$_{950}$-co-DIPAEMA)-1								
**9:1**	52.9	51.8	1.25	-433	-	-	-	-
**8:2**	52.8	51.8	1.26	-438	-	-	-	-
**7:3**	52.8	51.8	1.22	-322	-	-	-	-
**6:4**	52.8	51.9	1.20	-347	-	-	-	-
**5:5**	52.8	51.8	1.44	-255	-	-	-	-
DSPC:P(OEGMA$_{950}$-co-DIPAEMA)-2								
**9:1**	52.9	51.8	1.24	-420	-	-	-	-
**8:2**	52.8	51.8	1.29	-409	-	-	-	-
**7:3**	52.7	51.8	1.25	-318	-	-	-	-
**6:4**	52.7	51.8	1.51	-292	-	-	-	-
**5:5**	52.8	51.8	1.51	-169	-	-	-	-

[1] Tonset : temperature at which the thermal event starts
[2] T : temperature at which heat capacity (ΔCp) at constant pressure is maximum
[3] ΔT½ : half width at half peak height of the transition
[4] ΔH : transition enthalpy normalized per mol of lipid system
m : main transition, **s** : secondary transition

Table S4. Calorimetric parameters of DSPC:P(OEGMA$_{950}$-co-DIPAEMA hybrid bilayers during heating in dilution medium HCl 0.1 N (pH 1.2).

Weight ratio	$T_{onset,m}$ (°C)[1]	T_m (°C)[2]	$\Delta T_{½,m}$ (°C)[3]	ΔH_m (J/mol)[4]	$T_{onset,s}$ (°C)	T_s (°C)	$\Delta T_{½,s}$ (°C)	ΔH_s (J/mol)
				**DSPC:P(OEGMA$_{950}$-co-DIPAEMA)-1**				
**9:1**	56.9	59.0	1.99	201	-	-	-	-
**8:2**	55.4	57.8	2.67	288	-	-	-	-
**7:3**	54.7	56.7	1.97	328	-	-	-	-
**6:4**	54.1	56.1	2.07	284	-	-	-	-
**5:5**	54.2	55.8	1.75	170	-	-	-	-
				**DSPC:P(OEGMA$_{950}$-co-DIPAEMA)-2**				
**9:1**	55.28	57.8	3.38	269	-	-	-	-
**8:2**	54.75	56.8	3.29	370	-	-	-	-
**7:3**	52.95	54.9	1.91	341	-	-	-	-
**6:4**	53.51	55.1	1.73	330	-	-	-	-
**5:5**	53.26	55.0	1.69	299	-	-	-	-

[1] Tonset : temperature at which the thermal event starts
[2] T : temperature at which heat capacity (ΔCp) at constant pressure is maximum
[3] ΔT½ : half width at half peak height of the transition
[4] ΔH : transition enthalpy normalized per mol of lipid system
m : main transition, **s** : secondary transition

Table S5. Calorimetric parameters of DSPC:P(OEGMA$_{950}$-co-DIPAEMA hybrid bilayers during cooling in dilution medium HCl 0.1 N (pH 1.2).

Weight ratio	$T_{onset,m}$ (°C)[1]	T_m (°C)[2]	$\Delta T_{½,m}$ (°C)[3]	ΔH_m (J/mol)[4]	$T_{onset,s}$ (°C)	T_s (°C)	$\Delta T_{½,s}$ (°C)	ΔH_s (J/mol)
				**DSPC:P(OEGMA$_{950}$-co-DIPAEMA)-1**				
**9:1**	57.7	55.3	3.37	-552	-	-	-	-
**8:2**	56.7	54.0	3.42	-390	-	-	-	-
**7:3**	55.3	53.3	2.47	-358	-	-	-	-
**6:4**	54.8	52.8	2.47	-310	-	-	-	-
**5:5**	53.9	52.4	1.68	-131	-	-	-	-
				**DSPC:P(OEGMA$_{950}$-co-DIPAEMA)-2**				
**9:1**	57.8	54.1	4.58	-580	-	-	-	-
**8:2**	57.0	53.5	3.90	-478	-	-	-	-
**7:3**	53.5	51.8	2.37	-349	-	-	-	-
**6:4**	53.1	51.8	1.98	-331	-	-	-	-
**5:5**	53.0	51.6	2.16	-304	-	-	-	-

[1] Tonset : temperature at which the thermal event starts
[2] T : temperature at which heat capacity (ΔCp) at constant pressure is maximum
[3] ΔT½ : half width at half peak height of the transition
[4] ΔH : transition enthalpy normalized per mol of lipid system
m : main transition, **s** : secondary transition

Table S6. Calorimetric parameters of DSPC:DOPC:P(OEGMA$_{950}$-co-DIPAEMA-1 hybrid bilayers during heating.

Weight ratio	$T_{onset,m}$ (°C)[1]	T_m (°C)[2]	$\Delta T_{½,m}$ (°C)[3]	ΔH_m (J/mol)[4]	$T_{onset,s}$ (°C)	T_s (°C)	$\Delta T_{½,s}$ (°C)	ΔH_s (J/mol)
			Lipids: DSPC:DOPC (9:1 *weight ratio*)					
**9:1**	52.0	54.3	2.10	329	-	-	-	-
**7:3**	50.2	52.8	2.49	237	-	-	-	-
**5:5**	50.8	53.3	2.67	233	-	-	-	-
			Lipids: DSPC:DOPC (4:6 *weight ratio*)					
**9:1**	37.2	42.1	8.00	180	-	-	-	-
**7:3**	36.4	42.3	5.67	53	-	-	-	-
**5:5**	36.1	42.8	6.01	44	-	-	-	-

[1] Tonset : temperature at which the thermal event starts
[2] T : temperature at which heat capacity (ΔCp) at constant pressure is maximum
[3] ΔT½ : half width at half peak height of the transition
[4] ΔH : transition enthalpy normalized per mol of lipid system
m : main transition, **s** : secondary transition

Table S7. Calorimetric parameters of DSPC: DOPC: P(OEGMA$_{950}$-co-DIPAEMA-1 hybrid bilayers during cooling.

Weight ratio	$T_{onset,m}$ (°C)[1]	T_m (°C)[2]	$\Delta T_{½,m}$ (°C)[3]	ΔH_m (J/mol)[4]	$T_{onset,s}$ (°C)	T_s (°C)	$\Delta T_{½,s}$ (°C)	ΔH_s (J/mol)
			Lipids: DSPC:DOPC (9:1 *weight ratio*)					
**9:1**	52.5	51.5	2.15	-361	-	-	-	-
**7:3**	51.7	50.3	2.50	-247	-	-	-	-
**5:5**	52.5	50.7	2.60	-278	-	-	-	-
			Lipids: DSPC:DOPC (4:6 *weight ratio*)					
**9:1**	43.6	39.3	8.73	-235	-	-	-	-
**7:3**	42.0	39.5	6.03	-74	-	-	-	-
**5:5**	42.2	39.8	5.77	-59	-	-	-	-

[1] Tonset : temperature at which the thermal event starts
[2] T : temperature at which heat capacity (ΔCp) at constant pressure is maximum
[3] ΔT½ : half width at half peak height of the transition
[4] ΔH : transition enthalpy normalized per mol of lipid system
m : main transition, **s** : secondary transition

Table S8. Calorimetric parameters of DSPC:DOPC:P(OEGMA$_{950}$-co-DIPAEMA-2 hybrid bilayers during heating.

Weight ratio	$T_{onset,m}$ (°C)[1]	T_m (°C)[2]	$\Delta T_{½,m}$ (°C)[3]	ΔH_m (J/mol)[4]	$T_{onset,s}$ (°C)	T_s (°C)	$\Delta T_{½,s}$ (°C)	ΔH_s (J/mol)
**Lipids: DSPC:DOPC (9:1 *weight ratio*)**								
**9:1**	49.2	52.5	3.93	294	-	-	-	-
**7:3**	49.5	52.5	2.94	221	-	-	-	-
**5:5**	50.1	52.9	2.48	187	-	-	-	-
**Lipids: DSPC:DOPC (4:6 *weight ratio*)**								
**9:1**	35.7	42.0	5.85	75	-	-	-	-
**7:3**	38.9	43.7	5.06	59	-	-	-	-
**5:5**	-	-	-	-	-	-	-	-

[1] Tonset : temperature at which the thermal event starts
[2] T : temperature at which heat capacity (ΔCp) at constant pressure is maximum
[3] ΔT½ : half width at half peak height of the transition
[4] ΔH : transition enthalpy normalized per mol of lipid system
m : main transition, **s** : secondary transition

Table S9. Calorimetric parameters of DSPC:DOPC:P(OEGMA$_{950}$-co-DIPAEMA-2 hybrid bilayers during cooling.

Weight ratio	$T_{onset,m}$ (°C)[1]	T_m (°C)[2]	$\Delta T_{½,m}$ (°C)[3]	ΔH_m (J/mol)[4]	$T_{onset,s}$ (°C)	T_s (°C)	$\Delta T_{½,s}$ (°C)	ΔH_s (J/mol)
**Lipids: DSPC:DOPC (9:1 *weight ratio*)**								
**10:0**	51.8	49.3	3.38	-350	-	-	-	-
**9:1**	52.6	49.8	3.35	-363	-	-	-	-
**7:3**	52.0	49.8	3.46	-267	-	-	-	-
**5:5**	52.0	50.4	3.12	-196	-	-	-	-
**Lipids: DSPC:DOPC (4:6 *weight ratio*)**								
**10:0**	43.2	40.5	6.25	-111	-	-	-	-
**9:1**	42.0	39.4	4.82	-68	-	-	-	-
**7:3**	46.9	41.1	7.76	-191	-	-	-	-
**5:5**	-	-	-	-	-	-	-	-

[1] Tonset : temperature at which the thermal event starts
[2] T : temperature at which heat capacity (ΔCp) at constant pressure is maximum
[3] ΔT½ : half width at half peak height of the transition
[4] ΔH : transition enthalpy normalized per mol of lipid system
m : main transition, **s** : secondary transition

Table S10. Physicochemical properties of hybrid systems incorporating co-polymer P(OEGMA$_{950}$-DIPAEMA)-1 at 25◦C and in different pH media.

Sample	*Weight Ratio*	*Medium*	*I (kHz)*	*R_h (nm)*	*PDI*
DSPC:1	9:1	HCL 0.1N [pH 1.2]	3790	54	0,30
		WFI [pH 5.5]	4210	62	0,31
		PBS [pH 7.4]	14645	101	0,29
DSPC:1	7:3	HCL 0.1N [pH 1.2]	2090	44	0,33
		WFI [pH 5.5]	21645	103	0,20
		PBS [pH 7.4]	10500	110	0,35
DSPC:1	5:5	HCL 0.1N [pH 1.2]	659	(i) 19 (50%), (ii) 52 (50%)	0,43
		WFI [pH 5.5]	3023	58	0,31
		PBS [pH 7.4]	3394	79	0,34
DSPC:DOPC:1	9:1	HCL 0.1N [pH 1.2]	5300	493	0,44
		WFI [pH 5.5]	4230	95	0,40
		PBS [pH 7.4]	18097	181	0,44
DSPC:DOPC:1	7:3	HCL 0.1N [pH 1.2]	5180	268	0,40
		WFI [pH 5.5]	16419	99	0,27
		PBS [pH 7.4]	30700	150	0,31
DSPC:DOPC:1	5:5	HCL 0.1N [pH 1.2]	2752	144	0,39
		WFI [pH 5.5]	192	57	0,52
		PBS [pH 7.4]	531	(i) 17 (40%), (ii) 161 (60%)	0,53

Table S11. Physicochemical properties of hybrid systems incorporating co-polymer P(OEGMA$_{950}$-DIPAEMA)-2 at 25◦C and in different pH media.

Sample	*Weight Ratio*	*Medium*	*I (kHz)*	*R_h (nm)*	*PDI*
DSPC:2	9:1	HCL 0.1N [pH 1.2]	6000	78	0.27
		WFI [pH 5.5]	1087	(i) 16(23%), (ii) 58(76%)	0.42
		PBS [pH 7.4]	3390	87	0.35
DSPC:2	7:3	HCL 0.1N [pH 1.2]	1568	37	0.32
		WFI [pH 5.5]	413	23	0.46
		PBS [pH 7.4]	873	50	0.42
DSPC:2	5:5	HCL 0.1N [pH 1.2]	1100	(i) 12(8%), (ii) 44(64%), (iii)392(28%)	0.47
		WFI [pH 5.5]	499	33	0.27
		PBS [pH 7.4]	1077	(i) 46(87%), (ii) 1900(20%)	0.48
DSPC:DOPC:2	9:1	HCL 0.1N [pH 1.2]	6980	255	0.33
		WFI [pH 5.5]	3970	69	0.28
		PBS [pH 7.4]	25032	250	0.47
DSPC:DOPC:2	7:3	HCL 0.1N [pH 1.2]	4850	435	0.43
		WFI [pH 5.5]	4730	i)17(8%), ii)94(92%)	0.33
		PBS [pH 7.4]	16452	183	0.38
DSPC:DOPC:2	5:5	HCL 0.1N [pH 1.2]	4980	299	0.47
		WFI [pH 5.5]	493	i)18 (55%), ii)108 (44%)	0.46
		PBS [pH 7.4]	1142	i)15 (19%), ii)108 (80%)	0.49

Table S12. Physicochemical properties of hybrid systems incorporating co-polymer P(OEGMA$_{950}$-DIPAEMA)-1 at different temperatures and in water for injection dispersion medium (WFI).

Sample	*Weight Ratio*	*T (◦C)*	*I (kHz)*	R_h *(nm)*	*PDI*
DSPC:1	9:1	25	4210	62	0.31
		37	3660	66	0.31
		60	1529	42	0.33
DSPC:1	7:3	25	21645	103	0.20
		37	11516	86	0.16
		60	1481	49	0.15
DSPC:1	5:5	25	3023	58	0.31
		37	1394	55	0.29
		60	461	31	0.26
DSPC:DOPC:1	9:1	25	4230	95	0.40
		37	4260	97	0.39
		60	393	i)22 (70%), ii)158 (30%)	0.39
DSPC:DOPC:1	7:3	25	16419	99	0.27
		37	7430	74	0.41
		60	1671	45	0.05
DSPC:DOPC:1	5:5	25	192	57	0.52
		37	214	50	0.51
		60	185	53	0.50

Table S13. Physicochemical properties of hybrid systems incorporating co-polymer P(OEGMA$_{950}$-DIPAEMA)-2 at different temperatures and in water for injection dispersion medium (WFI).

Sample	*Weight Ratio*	*T (◦C)*	*I (kHz)*	*R_h (nm)*	*PDI*
DSPC:2	9:1	25	1087	(i) 16(23%), (ii) 58(76%)	0.42
		37	1148	(i) 20(33%), (ii) 82(66%)	0.39
		60	966	(i) 14(14%), (ii) 42(86%)	0.36
DSPC:2	7:3	25	413	23	0.46
		37	394	24	0.43
		60	586	27	0.28
DSPC:2	5:5	25	499	33	0.27
		37	586	27	0.28
		60	461	31	0.26
DSPC:DOPC:2	9:1	25	3970	69	0.28
		37	3730	(i) 26(18%), (ii) 81(82%)	0.31
		60	2503	47	0.18
DSPC:DOPC:2	7:3	25	4730	(i) 17(8%), (ii) 94(92%)	0.33
		37	3990	(i) 30(22%), (ii) 102(78%)	0.33
		60	2010	48	0.04
DSPC:DOPC:2	5:5	25	493	(i) 18(55%), (ii) 108(44%)	0.46
		37	454	24	0.37
		60	400	35	0.37

References

78. Selianitis, D and Pispas, S. P(MMA-co-HPMA)-b-POEGMA copolymers: synthesis, micelle formation in aqueous media and drug encapsulation. Polym Int. 2021;70:1508-1522. https://doi.org/10.1002/pi.6229.
79. Skandalis A and Pispas, S. PDMAEMA-b-PLMA-b-POEGMA triblock terpolymers via RAFT polymerization and their self-assembly in aqueous solutions. Polym Chem. 2017;8:4538–4547. https://doi.org/10.1039/C7PY00905D.

80. Skandalis A, Pispas S. PLMA-b-POEGMA amphiphilic block copolymers: Synthesis and self-assembly in aqueous media. Polym Chem. 2017;55: 155-163. https://doi.org/10.1002/pola.28379.
81. Góis JR, Rocha N, Popov AV, Guliashvili T, Matyjaszewski K, Serra AC, & Coelho JFJ. Synthesis of well-defined functionalized poly(2-(diisopropylamino)ethyl methacrylate) using ATRP with sodium dithionite as a SARA agent. Polym. Chem. 2014;5(12):3919–3928. https://doi.org/10.1039/C4PY00042K.
82. Avanti Polar Lipids. Available online: https://avantilipids.com/product/850365 (accessed 17 June 2022) [supporting information]
83. Avanti Polar Lipids. Available online: https://avantilipids.com/product/850375 (accessed 17 June 2022) [supporting information]
